# Oral microbiota dysbiosis in pediatric patients undergoing treatment for acute lymphoid leukemia a preliminary study

**DOI:** 10.1590/1678-4685-GMB-2023-0359

**Published:** 2025-05-16

**Authors:** André Vieira Souza, Leonardo Vinícius Barbosa, Alejandra Adriana Cardoso de Castro, Edna Kakitani Carboni, Flora Mitie Watanabe, Roberto Rossati, Libera Maria Dalla Costa, Dany Mesa, Cleber Machado-Souza

**Affiliations:** 1 Faculdades Pequeno Príncipe, Curitiba, PR, Brazil.; 2 Instituto de Pesquisa Pelé Pequeno Príncipe, Curitiba, PR, Brazil.; 3 Hospital Pequeno Príncipe, Curitiba, PR, Brazil.

**Keywords:** Acute lymphoblastic leukemia, human microbiome, microbiome, microbial community structure, mouth mucosa, mucositis

## Abstract

Acute lymphoblastic leukemia (ALL) stands out as the most prevalent neoplasm during childhood, characterized by the rapid production of abnormal lymphoid cells. Chemotherapy administered to these patients may induce a substantial imbalance in the oral microbiota. A prospective pediatric study encompassing a control group (without ALL) and ALL patients at two treatment stages (pre-induction and consolidation) was conducted. Clinical and laboratory data were meticulously collected. Moreover, DNA from saliva samples was extracted for *16S rRNA* sequencing. Clinical data revealed a heightened incidence of oral mucositis during the consolidation phase. Analysis of alpha biodiversity (observed taxa) exhibited a significant reduction in bacterial richness among patients in the consolidation phase. Network analysis identified key taxa during this phase, namely *Neisseria flavescens*, *Prevotella melaninogenica* and *Porphyromona*s. The findings underscore the substantial impact of ALL treatment on the oral microbiota composition, indicating diminished bacterial diversity and an elevated prevalence of oral mucositis.

## Introduction

Acute lymphoblastic leukemia (ALL) is a hematological neoplasm characterized by the exacerbated proliferation of blasts in the bone marrow and mainly affects children aged 2 to 15 years (Béné, 2005; Miranda et al., 2018). Patients with hematological and/or oncological diseases usually present harmful oral manifestations as a result of the intense immunosuppression obtained through chemotherapy treatment. Pathogenic bacterial growth negatively affects the oral mucosa and, thus, the associated immune system (Fattizzo et al., 2021), indicating that a healthy microbiome not only provides nutrition and a natural barrier but also influences the outcome of medical treatment (Blijlevens *et al.,* 2000). Thus, especially in compromised individuals, such as those with hematological malignancies, the oral environment can be assisted throughout the treatment period to promote not only oral health but also general health (Santos and Soares, 2012). 

In health, there is an ecological balance between the human host and the microorganisms that colonize mucosal surfaces. Pediatric patients undergo chemotherapy as part of their treatment, which alters the resident microbiota (Oldenburg et al., 2021). The cytotoxic effects of these treatments lead to greater immunosuppression, causing complications (febrile neutropenia and infections), thus bringing changes in the ecosystem that can disrupt the ecological balance of the oral and gastrointestinal microbiota (Rajagopala et al., 2019). 

Understanding the dysbiosis pattern in the oral microbiome can help identify specific microbial agents that act as biomarkers in ALL patients. Thus, the aim of this study was to describe the shifts in the oral microbiota in pediatric patients with ALL undergoing antineoplastic therapy.

## Subjects and Methods

### Study population and oral clinical condition

From September 2020 to December 2021 eight pediatric patients (from 4 to 11 years old) and diagnosed with ALL were admitted for treatment in the oncology sector of the Hospital Pequeno Príncipe (HPP) located in Curitiba, Paraná - Brazil and included in this project. The diagnosis of ALL followed morphological, cytochemical, immunophenotyping and cytogenetic international criteria (Pui et al., 2018), These patients were treated according to the RE-LLA-2005 protocol (Pedrosa and Lins, 2002). The control group (four patients from 4 to 11 years old) attended routine dental physical examination at the dentistry service of HPP and did not present onco-hematological disease. This study was approved by the research ethics committee of HPP (Protocol Number: 3.836.067/2020). Three patients’ groups were organized in the present study:

1. pre-induction: Eight ALL patients in the phase prior to induction (D0 to D5).
2. consolidation: Eight ALL patients in the consolidation phase (D40 to D45).
3. control: Four patients without onco-hematological disease.

Demographic, clinical (medical and dental history) and laboratory data were obtained from HPP’s electronic medical record and by direct interview (Table 1). Microbiota samples and intraoral physical examination was performed. At the same time as the microbiota collection, the results of routine laboratory tests (marker dosages) of the patients were accessed from the internal system. The intraoral physical examination of all groups was performed after acceptance and before the microbioma samples collection.

**Table 1:** Characteristics of the study participants from Hospital Pequeno Príncipe 2021.

Variables	ALL (n=8)	CONTROL (n=4)	p-value
Sex**			
Male	4 (50.0)	2 (50.0)	1.000^b^
Female	4 (50.0)	2 (50.0)	
Age*	4.7±6.0	7.2±10.9	0.142^a^
Neutropenia**			
Yes	7 (87.5)	0 (0.0)	0.001^b^
No	1 (12.5)	4 (100.0)	
Mucosite**			
Grade 0	0 (00.0)	4 (100.0)	0.133^b^
Grade I	2 (20.0)	0 (0.00)	
Grade II	4 (40.0)	0 (0.00)	
Grade III	2 (20.0)	0 (0.00)	
Disfagy**			
Yes	5 (50.0)	0 (0.0)	0.006^b^
No	3 (30.0)	4 (100.0)	
Biofilm accumulation**			
Yes	6 (60.0)	0 (00.0)	0.006^b^
No	2 (20.0)	4 (100.0)	
Gum Inflamation**			
Yes	5 (50.0)	0 (00.0)	0.005^b^
No	3 (30.0)	4 (100.0)	
Changes in oral mucosa**			
Yes	5 (50.0)	0 (00.0)	0.006^b^
No	3 (30.0)	4 (100.0)	

Note. *Mean and standard deviation; **number and frequency; ^a^Mann-Whitney U test; ^b^Pearson’s Chi-square test.

The assessment of the patient’s oral condition was performed through a unique dentistry and evaluated (i) Dental biofilm: a thin adherent layer formed by bacteria and other substances from saliva, accumulating on teeth and other oral surfaces. (ii) Gingival inflammation: known as gingivitis, results from bacterial plaque on teeth, leading to red, swollen, and sensitive gums with bleeding during brushing (Franceschini et al., 2003). (iii) Mucositis: an inflammation of the mucosa, the moist lining that covers the mouth and gastrointestinal tract. On the other hand, blood leukocyte profile was obtained by leukocytes, neutrophils, lymphocytes, monocytes, and platelets traditional counts (Sonis, 2004). 

### Collection of biological material

Biological samples for the analysis of the oral microbiota were collected at two different times during the ALL treatment (pre-induction and consolidation). Patients in the control group collected only one time after they accepted to participate in the research. Oral rinse with 5 ml of 0.9% saline solution for 1 minute was collected from each patient and immediately frozen in liquid nitrogen and transferred to a -80 °C freezer at the central laboratory until the time of extraction of bacterial genetic material (Lim et al., 2017).

### DNA extraction, sequencing, and bioinformatic analysis

Around 150 mg of oral rinse was sampled for DNA extraction using the ZymoBIOMICS DNA^®^ kit, according to the manufacturer’s instructions. The concentrations and quality of the extracted DNA were measured using a NanoDrop spectrophotometer (Nanodrop Technologies, Wilmington, DE, USA). The integrity of the DNA was also confirmed by electrophoresis in a 1% agarose gel with 1×TAE buffer (Klindworth et al., 2021). 

PCR primers (F515/R806) were used to amplify the V4 region of the 16S rRNA gene. PCR was performed at 94 ºC for 3 min to denature the DNA, followed by 28 cycles at 94 ºC for 45 s, 50 ºC for 60 s, and 72 *°*C for 90 s, with a ﬁnal extension of 10 min at 72 ºC to ensure complete ampliﬁcation (Klindworth et al., 2021). The amplicons were quantified with Qubit using HS dsDNA kit (Invitrogen, Carlsbad, CA, USA), diluted to 500 pM, and pooled. Next, 16 pM of pooled DNA was sequenced using MiSeq reagent 500V2 (Illumina, San Diego, CA, USA). Sequencing was performed using a MiSeq^®^ sequencer (Illumina), obtaining paired reads of 250 bp, as previously described (Caporaso et al., 2011). Four samples of (mock microbiota) of ZymoBIOMICS^®^ Microbial Community Standard kit were used as positive control.

Raw reads quality was checked using FastQC and when necessary, reads were trimmed using Trimmomatic (Bolger et al., 2014). Sequencing reads were analyzed with the QIIME2 pipeline (Bolyen et al., 2019). Sequences were sorted according to quality and chimeras were then removed from the dataset using DADA2 algorithm. Following, good quality sequences were clustered into amplicon sequence variant (ASV) using the DADA2 algorithm inserted in the QIIME2 program (Bolger *et al.,* 2014). Furthermore, taxonomic assignment was performed using the SILVA 138 database, release 2020 and the q2-vsearch algorithm of the QIIME2 program (Yilmaz et al., 2013; Callahan et al., 2016). The reads output was normalized to 9,000 per sample, allowing alpha and beta diversity comparisons between groups (Rognes et al., 2016; Bolyen *et al*., 2019). Detailed information of reads per sample can be found in Table S1 and rarefaction curves (Figure S1).

### Pearson correlation and network analysis

Blood leukocyte profile of patients (leukocytes, neutrophils, lymphocytes, monocytes, and platelets) was correlated with the taxa that showed significant differences between the treatments. Pearson’s correlation was calculated and plotted using the *corrplot* and *RColorBrewer* packages included in R software, statistically significant results (*P*<0.05) were highlighted with asterisk in the plots. Additionally, to identify key taxa at the time of consolidation, we constructed a network using Cytoscape program with the strongest positive and negative correlations (between 0.6-1) that were also statistically significant (*P*<0.05).

### Data accessibility

The dataset was submitted to the National Center for Biotechnology Information (NCBI) under the BioSample accession code SAMN25696387.

### Statistical analysis

Bacterial taxa abundance comparisons between treatments were conducted in the STAMP software using Welch’s t-test (*P*<0.05) and Bonferroni’s correction (Caporaso et al., 2011). Alpha diversity analysis (number of features) was compared between collected times using Wilcoxon’s test (*P*<0.05). Only statistically significant results were reported (*P*< 0.05). Beta biodiversity analysis, represented by principal component analysis (PCA) were done using a table with bacterial taxa abundance and the STAMP software.

## Results

At the time of diagnosis, patients with ALL had a minimum age of four years (4.7±6.0), while the control group’s age was seven years (7.2±10.9). Both groups had an equal distribution between male and female genders. The ALL patients showed a high prevalence of neutropenia (7/8) and notable cases of gingival inflammation (5/8) accompanied by visible biofilm accumulation (6/8). Concerning mucositis, some patients experienced grade III mucositis (2/8), but the majority had grade II mucositis (4/8), leading to a significant occurrence of dysphagia (5/8). In the control group, no clinical alterations were observed.

**Figure 1:**
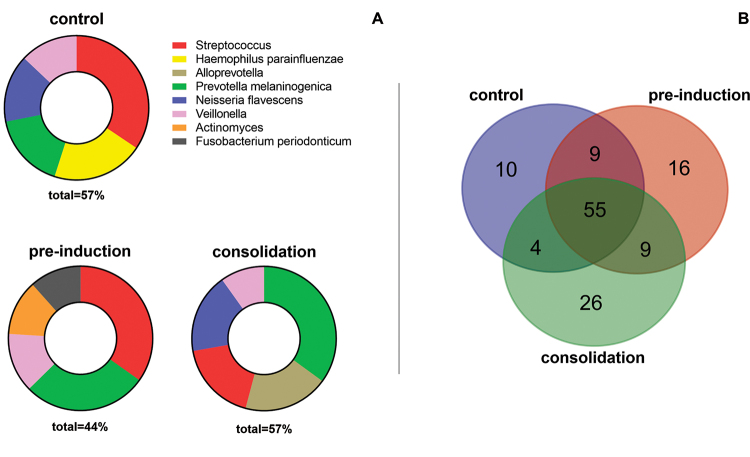
The most abundant taxa per treatment. **(A)** Relative frequency of the most abundant taxa per treatment. (**B)** Venn diagram representing the core microbiota of the communities, taxa at genera and species level were used in the construction of the diagram. 55 taxa were identified as the core microbiota, and 10 taxa were exclusive to the control group.

**Figure 2:**
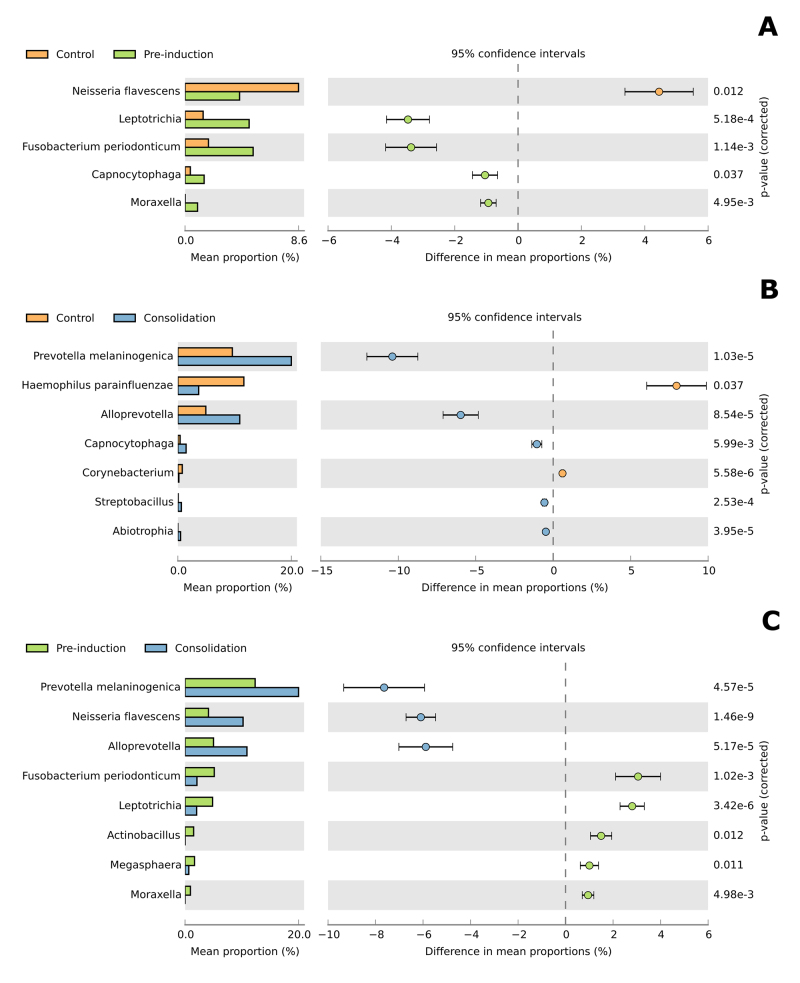
Statistical comparison of relative abundance between taxa in the treatments. Significant increases of *Leptotrichia*, *Fusobacterium periodonticum*, and *Capnocytophaga* were observed in the pre-induction phase compared to the control group (**A**). Significant increases of *Prevotella melaninogenica*, *Alloprevotella*, and *Capnocytophaga* were observed in the consolidation phase compared to the control group (**B**). A significant increase of *Prevotella melaninogenica*, *Neisseria flavescens*, and *Alloprevotella* was observed between the pre-induction and consolidation phases (**C**).

The analysis of the bacterial community revealed distinct patterns in the control group and the pre-induction and consolidation phases of ALL treatment. In the control group, the most abundant taxa were the genera *Streptococcus, Veillonella*, and the species *Haemophilus parainfluenzae*, *Prevotella melaninogenica*, and *Neisseria flavescens* (Figure 1A). In the pre-induction phase, the genera *Streptococcus, Veillonella, Actinomyces*, and the species *Prevotella melaninogenica* and *Fusobacterium periodonticum* were more prevalent (Figure 1A). During the consolidation phase, the genera *Streptococcus, Veillonella, Alloprevotell*a, and the species *Prevotella melaninogenica* and *Neisseria flavescens* dominated the microbial composition (Figure 1A). Additional analysis of the core microbiota showed taxa common to all groups and those exclusive to each treatment (Figure 1B). Thus, ten exclusive taxa were observed in the control group*: Pirellula, Kurthia, Fretibacterium, Bifidobacterium, Desulfovibrio, Caproiciproducens, Anaerofilum, Anaerovorax, Clostridium sensu stricto 11*, and *Pseudopropionibacterium*. In the pre-induction phase, 16 unique taxa were identified, and in the consolidation phase, 26 unique taxa were identified (Table S2). Statistical comparison of relative abundance between taxa in the treatments showed a significant increase of *Leptotrichia*, *Fusobacterium periodonticum* and *Capnocytophaga* in the pre-induction phase when compared to the control group (Figure 2A). Significant increase of *Prevotella melaninogenica*, *Alloprevotell*a and *Capnocytophaga* in the consolidation phase when compared to the control group (Figure 2B). Between the pre-induction and consolidation, a significant increase of *Prevotella melaninogenica*, *Neisseria flavescens* and *Alloprevotell*a were observed in the consolidation phase (Figure 2C). 

**Figure 3:**
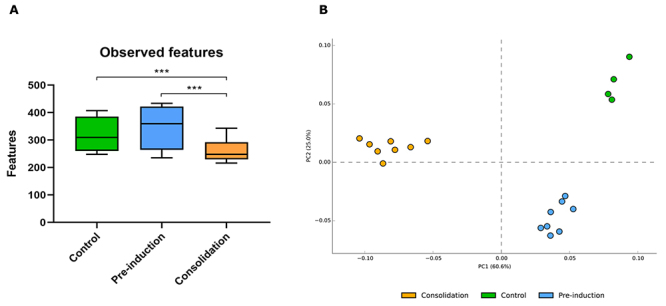
Richness of communities. **(A)** Number of *taxa* identified per treatment. Bars in the plot represent the mean and standard deviation of the observed taxa in each treatment. Asterisks indicate the relationships that showed significant differences by Wilcoxon test. (p < 0.05). **(B)** PCA plot representing the grouping of samples by treatment. Dots of the same color indicate samples from the same treatment.

On the other hand, the analysis of alpha biodiversity (observed taxa) showed a significant decrease in bacterial richness in the patients of the consolidation group compared to the pre-induction and control group (Figure 3A). Additionally, beta biodiversity analysis, represented by principal component analysis (PCA), showed the grouping of samples, suggesting a marked differentiation of bacterial communities by treatment. Thus, microbiota changes between the control and the pre-induction group were smaller (PC2:25%) compared to microbiota changes between the control and the consolidation group (PC1:60%). This result suggests that the consolidation phase of the treatment led to more significant changes in the composition of the oral microbiota when compared to patients of the control group (Figure 3B).

**Figure 4:**
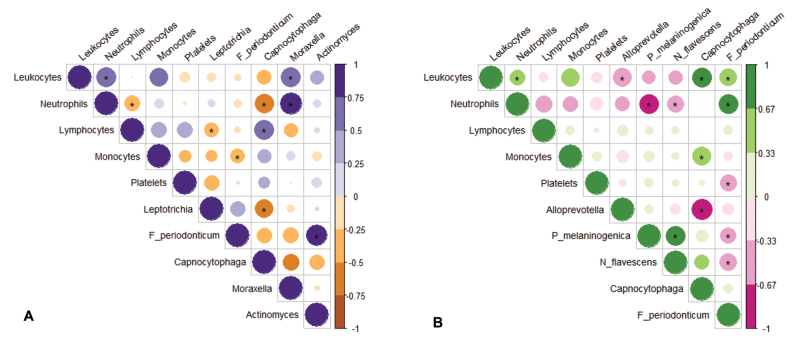
Pearson’s correlation between microbiota and leukocyte profile. **(A)** Pre-induction correlation matrix. **(B)** Consolidation correlation matrix. The figure represents the correlations between two variables on a scale from 1 to -1, where negative values indicate negative relationships. The size of the circles in the matrix represents the scale of the correlations, and asterisks indicate relationships that present significant differences.

Pearson’s correlation between microbiota and leukocyte profile in the pre-induction group showed significant and negative relationships between neutrophils and *Capnocytophaga*; lymphocytes *and Leptotrichia*. On the other hand, positive relationships were identified between neutrophils and *Moraxela*; lymphocytes and *Capnocytophag*a; leukocytes and *Moraxella* (Figure 4A). In the consolidation, the following significant and negative correlations were identified: between neutrophils and *Neisseria flavescens*; neutrophils and *Prevotella melaninogenica*; leukocytes and *Alloprevotella*. On the other hand, positive relationships were identified between leukocytes and *Capnocytophaga*; leukocytes and *Fusobacterium Periodonticum*; neutrophils and *Fusobacterium Periodonticum*; monocytes and *Capnocytophaga* (Figure 4B).

**Figure 5:**
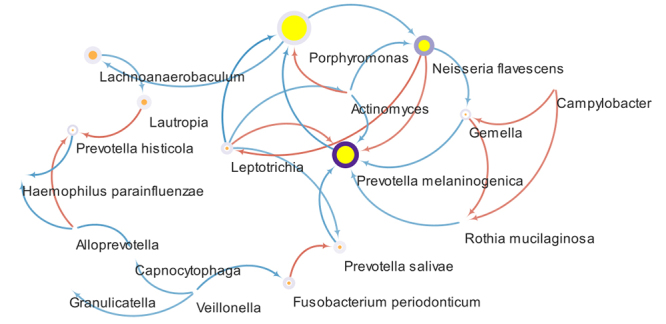
Network of consolidation. The figure represents the taxa and their relationships in the consolidation phase. Nodes represent taxa and the color of the circle around it represents its abundance in the community. The size of the nodes represents the taxon’s importance in the community given by the “Betweenness centrality” value. In yellow, the three key taxa of the community were highlighted. The edges between the nodes represent the correlations between two taxa, in red for positive correlations, and in blue for negative correlations. Only strong positive and negative correlations (between 0.6-1) and significant relationships (P < 0.05) are shown.

The results of the network analysis at the time of consolidation showed a community with 18 nodes, 29 edges, and an average number of neighbors of 3.2. The presence of some abundant taxa such as *Lactobacillus* and *Neisseria flavescens* was identified in the community. However, the three taxa responsible for the highest connectivity in the network were *Prevotella melaninogenica*, *Neisseria flavescense*, and *Porphyromonas*, considered as the key taxa at the time of consolidation (Figure 5). Of the 29 edges in the network, 19 were negative and 10 were positive. The strongest positive correlation (0.82) was between *Neisseria flavescens* and *Leptotrichia*. On the other hand, the strongest negative correlation (-0.90) was between *Leptotrichia* and *Porphyromonas*. Additionally, *Porphyromonas* showed other negative correlations with *Prevotella melaninogenica, Neisseria flavescens,* and *Lachnoanaerobaculum*, and only one positive correlation with *Actinomyces*.

## Discussion

The oral microbiota functions as a reservoir for planktonic-stage microorganisms that colonize/recolonize various habitats within the oral cavity. Studies by Griffen et al. (2012) and Wang et al. (2014) have highlighted *Streptococcus* as the most abundant genus in healthy oral microbiota, followed by *Prevotella, Veillonella, Neisseria,* and *Haemophilus,* aligning with the findings of this study. However, in immunocompromised patients, the oral microbiota can become opportunistic and pathogenic, leading to infections. This imbalance may manifest in leukemic symptoms such as pallor of the oral mucosa, gum discoloration, gingival petechiae, and ulcerative mucosal lesions, as observed in our clinical and blood profile results. Immunocompromised individuals are also more susceptible to bacterial and fungal infections (Kamasaki et al., 2005).

Our results indicate an increase in the abundance of the genus *Actinomyces* during the pre-induction phase and *Alloprevotella* during the consolidation phase among the five most abundant microorganisms. These shifts may signify a dysbiotic aspect across different chemotherapy treatment phases. *Streptococcus*, *Veillonella*, *Prevotella melaninogenica,* and *Neisseria flavescens* could be considered a central core.

*Actinomyces* may play a role in the bacterial community during the consolidation phase, forming a complex and diverse composition that might result from alterations in host defenses. In contrast, a less diverse commensal bacterial community during disease progression leaves individuals more susceptible to opportunistic diseases, impacting their quality of life; *Actinomyces* infections have been reported in cases of patients after hematopoietic stem cell transplantation (Carvalho et al., 2022). While oral infections by *Actinomyce*s are rare in immunocompetent individuals, they become more common in immunocompromised individuals, leading to considerable morbidity with atypical and variable presentations, potentially resulting in severe outcomes if not diagnosed early (Ali and Tanwir, 2012; Bolger et al., 2014).

In our study, the abundance of *Bacteroides* increased, and genus/species richness decreased in children with acute leukemia. Deficient neutrophils in childhood acute leukemia may lead to reduced energy intake and storage, resulting in a state of high energy consumption due to immune disturbance. This makes the body more susceptible to bacterial invasion and infections (Van Der Meulen, 2018). In the pre-induction and consolidation phases, we observed a positive and significant relationship between neutrophils and leukocytes in the pre-induction phase with the genus *Moraxella* and, in the consolidation phase, with the species *Fusobacterium periodonticum* (Wang et al., 2020). Moraxella is associated with respiratory tract infections, while *Fusobacterium periodonticum* is linked to deficient oral conditions (Cauduro et al., 1999; Silva, 2016). Different *Fusobacterium nucleatum* strains may differentially affect neutrophil function, indicating variations in their virulence properties (Kurgan *et al.,* 2016). In addition, data indicate that immunosuppression is also correlated with characteristic changes in the intestinal microbiota in humans, likely due to damages to gut-associated lymphoid (Lozupone et al., 2013). Thus, the intestinal microbiota seems to interact directly with the immune system of the host, stimulating local and systemic immune interactions (Sommer and Backhed, 2013). In this way, immunosuppression with cyclophosphamide led to significant decreases of several circulating leukocyte subsets in chickens and shifts in the intestinal microbiota (Mesa et al., 2020). 

*Fusobacterium periodonticum* seems to grow less efficiently in biofilms and exhibits a more condensed localization pattern in supra and subgingival biofilm models (Thurnheer et al., 2019). However, this behavior may be induced by the new conditions resulting from antineoplastic treatment in these patients (Cocivera et al., 2022). The correlation analysis of microbiota with laboratory data reinforces previous findings regarding the presence of dysbiosis throughout the treatment process of patients with ALL.

Regarding the oral microbiota network, until the submission of this work, no articles were found that conducted a network analysis of the oral microbiota. In Wang et al. (2020) study, the authors evaluated the richness and taxonomic composition of the oral microbiota. In the study by Hou et al. (2018), the oral microbiota in patients with nasopharyngeal carcinoma undergoing radiotherapy was evaluated. Interestingly, *Prevotella, Fusobacterium, Treponema, and Porphyromonas* showed synchronous dynamic variations in their abundances during radiotherapy, with peaks often coinciding with the onset of severe mucositis. Our network results align with this study, highlighting the significance of taxa *Prevotella melaninogenica, Neisseria flavescens, and Porphyromonas* during the consolidation phase of treatment and considering them as potential biological markers of a higher degree of oral mucositis, added to the drop in biodiversity and a significant increase in this phase when compared to the control group.

Further considerations about the three main microorganisms resulting from our network analysis are essential. *Porphyromonas,* in the consolidation phase, appears to be a potential marker in the observed dysbiosis during chemotherapy treatment phases (Oldenburg et al., 2021). It has been found to occupy other sites, such as the upper portion of the gastrointestinal tract and the colon. *In vitro* studies, *Porphyromonas* demonstrated the ability to invade human gingival fibroblasts as a form of protection against the effects of antimicrobial use. This adaptive ability as an intracellular parasite is noteworthy and could trigger changes in essential cellular mechanisms such as the cell cycle and cell death (Irshad et al., 2012). This capacity suggests that *Porphyromonas* might be involved in the recurrence of other types of neoplasms in patients after initial chemotherapy treatment phases (Wang et al., 2020).

Additionally, *Prevotella and Neiseria* were identified as significant microbial agents in the consolidation phase. Hsiao et al. (2018) listed three periodontal pathogenic species (*Prevotella tannerae, Fusobacterium nucleatum, and Prevotella* intermedia) associated with an increased risk of oral squamous cell carcinoma, further emphasizing the importance of considering these microorganisms as potential biomarkers of risk (Hsiao *et al.,* 2018).

Some studies suggest that non-pathobiont microorganisms, typically considered healthy-associated species like *Neisseria flavescens,* may contribute to carcinogenesis. *Neisseria flavescens*, along with *Haemophilus parainfluenzae*, have been found to induce cytotoxicity through intracellular infection. Thus, these three microorganisms could potentially be considered future targets for risk stratification in the development of secondary neoplasms (Baraniya et al., 2020). 

This study has some limitations, such as a small and heterogeneous cohort size. However, it represents the largest pediatric hospital in Brazil, making it a significant clinical cohort from the country. The COVID-19 pandemic significantly constrained sampling and patient inclusion in the study, both for sick and healthy individuals. Despite these limitations, the consistent and significant results related to oral microbiota contribute to a better understanding of bacterial shifts during different moments of antineoplastic treatment.

## Conclusions

The results indicate that ALL treatment can induce noteworthy alterations in the composition of the oral microbiota, characterized by decreased bacterial diversity and an elevated incidence of oral mucositis. Furthermore, specific bacterial taxa, including *Prevotella melaninogenica*, *Neisseria flavescens*, and *Porphyromonas,* emerged as pivotal components in the oral microbiota community during the consolidation phase of treatment, demonstrating a correlation with a heightened degree of oral mucositis. Understanding these changes is crucial for developing strategies to safeguard the oral health of patients undergoing ALL treatment.

Supplementary Material

The following online material is available for this article:

Table S1 - Reads per sample.

Table S2 - Core microbiota of the community.

Figure S1 - The figure S1 shows rarefaction curves for groups (Consolidation, Control and Pre-induction).
