# Supplementary material for: Oral microbiota dysbiosis in pediatric patients undergoing treatment for acute lymphoid leukemia a preliminary study
Source: Genet Mol Biol. 2025 May 16;48(2):e20230359. doi: 10.1590/1678-4685-GMB-2023-0359 (PMC12083558; doi:10.1590/1678-4685-GMB-2023-0359)
Supplement: Table S1 - [file 1415-4757-GMB-48-02-e20230359-s1.pdf]

Genetics and Molecular Biology
https://doi.org/10.1590/1678-4685-GMB-2023-0359

Supplementary Material to “Oral microbiota dysbiosis in pediatric patients undergoing treatment for acute lymphoid leukemia a preliminary study”

Table S1 – Reads per sample.

Sample-id	Input	Input filtered	Percentage (%) of input passed filter	Denoised	Merged	Percentage (%) of input merged	Non-chimeric	Percentage (%) of input non-chimeric
SC-P1	93485	73143	78.24	71758	65147	69.69	12855	13.75
SC-P2	134249	106627	79.42	105106	96296	71.73	16145	12.03
SC-P3	145996	83950	57.5	82589	74927	51.32	13662	9.36
SC-P4	238036	188662	79.26	186267	170865	71.78	25258	10.61
SMA-P1	54191	41835	77.2	40582	36509	67.37	10600	19.56
SMA-P2	114726	90168	78.59	88382	80997	70.6	17713	15.44
SMA-P3	49867	38672	77.55	37713	33883	67.95	9835	19.72
SMA-P4	112864	88183	78.13	86438	78775	69.8	17171	15.21
SMA-P5	177433	139745	78.76	137745	128709	72.54	23938	13.49
SMA-P6	183853	140209	76.26	137755	125748	68.4	24052	13.08
SMA-P7	87959	68571	77.96	66840	60241	68.49	15641	17.78
SMA-P8	64680	49965	77.25	48684	43526	67.29	12356	19.1
SMC-P1	201164	160948	80.01	159261	152433	75.78	23702	11.78
SMC-P2	58971	47226	80.08	46371	43562	73.87	9702	16.45
SMC-P3	81418	65451	80.39	64314	60920	74.82	11897	14.61
SMC-P4	71085	56423	79.37	55273	52052	73.23	10933	15.38
SMC-P5	72411	56863	78.53	55884	52838	72.97	11110	15.34
SMC-P6	56639	43912	77.53	42924	40291	71.14	9094	16.41
SMC-P7	108732	86713	79.75	85210	80152	73.72	14851	13.66
SMC-P8	77379	60730	78.48	59494	55889	72.23	11128	14.38
