# Supplementary material for: Oral microbiota dysbiosis in pediatric patients undergoing treatment for acute lymphoid leukemia a preliminary study
Source: Genet Mol Biol. 2025 May 16;48(2):e20230359. doi: 10.1590/1678-4685-GMB-2023-0359 (PMC12083558; doi:10.1590/1678-4685-GMB-2023-0359)
Supplement: Table S2 - [file 1415-4757-GMB-48-02-e20230359-s2.pdf]

Genetics and Molecular Biology
https://doi.org/10.1590/1678-4685-GMB-2023-0359

Supplementary Material to "Oral microbiota dysbiosis in pediatric patients undergoing treatment for acute lymphoid leukemia a preliminary study"

Table S2 – Core microbiota of the community. The table shows common taxa among different treatments.

Treatments	Total	Taxa
Pre-induction Consolidation Control	55	*Neisseria flavescens*
		Streptococcus
		Oribacterium
		Haemophilus parainfluenzae
		Peptococcus
		Prevotella 2
		Kingella
		Pseudomonas
		Selenomonas
		Prevotella melaninogenica
		Corynebacterium
		Fusobacterium periodonticum
		Butyrivibrio 2
		Bergeyella
		Candidatus Saccharimonas
		Olsenella
		Lautropia
		Alloprevotella
		Solobacterium
		Arcobacter
		Mycoplasma
		Selenomonas 3
		Tannerella
		Streptobacillus
		Capnocytophaga

Treatments	Total	Taxa
		Cardiobacterium
		Treponema 2
		Veillonella
		Clostridium sensu stricto 12
		Rothia mucilaginosa
		Prevotella 9
		Gemella
		Actinomyces
		Prevotella salivae
		Eubacterium nodatum
		uncultured bacterium
		Eubacterium brachy
		Granulicatella
		Parvimonas
		Lachnoanaerobaculum
		Catonella
		Megasphaera
		Succiniclasticum
		Peptostreptococcus
		Campylobacter
		Bacteroides
		Prevotella histicola
		Mogibacterium
		Lactococcus
		Atopobium
		Leptotrichia
		Porphyromonas
		Actinobacillus
		Lactobacillus
		Proteiniphilum
Pre-induction Control	9	*Empedobacter*
		Filifactor
		Acinetobacter
		Enterococcus
		Aggregatibacter

Treatments	Total	Taxa
		Novosphingobium
		Moraxella
		Weissella
		Enterobacter
Consolidation Control	4	*Clostridium sensu stricto 1*
		Sporanaerobacter
		Dysgonomonas
		Escherichia-Shigella
Pre-induction Consolidation	9	*Staphylococcus*
		Syntrophobacter
		Dialister
		Stenotrophomonas
		Terrisporobacter
		Streptomyces
		Abiotrophia
		Eubacterium yurii
		Achromobacter
Control	10	*Pirellula*
		Kurthia
		Fretibacterium
		Bifidobacterium
		Caproiciproducens
		Pseudopropionibacterium
		Anaerovorax
		Clostridium sensu stricto 11
		Anaerofilum
		Desulfovibrio
Pre-induction	16	*Brevundimonas*
		Eubacterium coprostanoligenes
		Shuttleworthia
		Iamia
		Delftia
		Johnsonella
		Leuconostoc
		Bacillus

Treatments	Total	Taxa
Consolidation	26	*Corynebacterium 1*<br>*Rubellimicrobium*<br>*Turicibacter*<br>*Butyricicoccus*<br>*Oceanivirga*<br>*Lutispora*<br>*Vibrio*<br>*Tyzzerella*<br>*Exilispira*<br>*Ruminococcus 2*<br>*Elusimicrobium*<br>*Clostridium sensu stricto 13*<br>*Desulfomicrobium*<br>*Christensenella*<br>*Methanolinea*<br>*Fluviicola*<br>*Prevotella 1*<br>*Pyramidobacter*<br>*Macellibacteroides*<br>*Methanosaeta*<br>*Thermomonas*<br>*Collinsella*<br>*Pseudonocardia*<br>*Sphingomonas*<br>*Mesotoga*<br>*Geobacter*<br>*Smithella*<br>*Rhizobium*<br>*Anaerotruncus*<br>*Saccharofermentans*<br>*Petrimonas*<br>*Klebsiella*<br>*Syntrophorhabdus*<br>*Spirochaeta 2*
