# Supplementary material for: Oral microbiota dysbiosis in pediatric patients undergoing treatment for acute lymphoid leukemia a preliminary study
Source: Genet Mol Biol. 2025 May 16;48(2):e20230359. doi: 10.1590/1678-4685-GMB-2023-0359 (PMC12083558; doi:10.1590/1678-4685-GMB-2023-0359)
Supplement: Figure S1 - [file 1415-4757-GMB-48-02-e20230359-s3.pdf]

Genetics and Molecular Biology
https://doi.org/10.1590/1678-4685-GMB-2023-0359

Supplementary Material to “Oral microbiota dysbiosis in pediatric patients undergoing treatment for acute lymphoid leukemia a preliminary study”

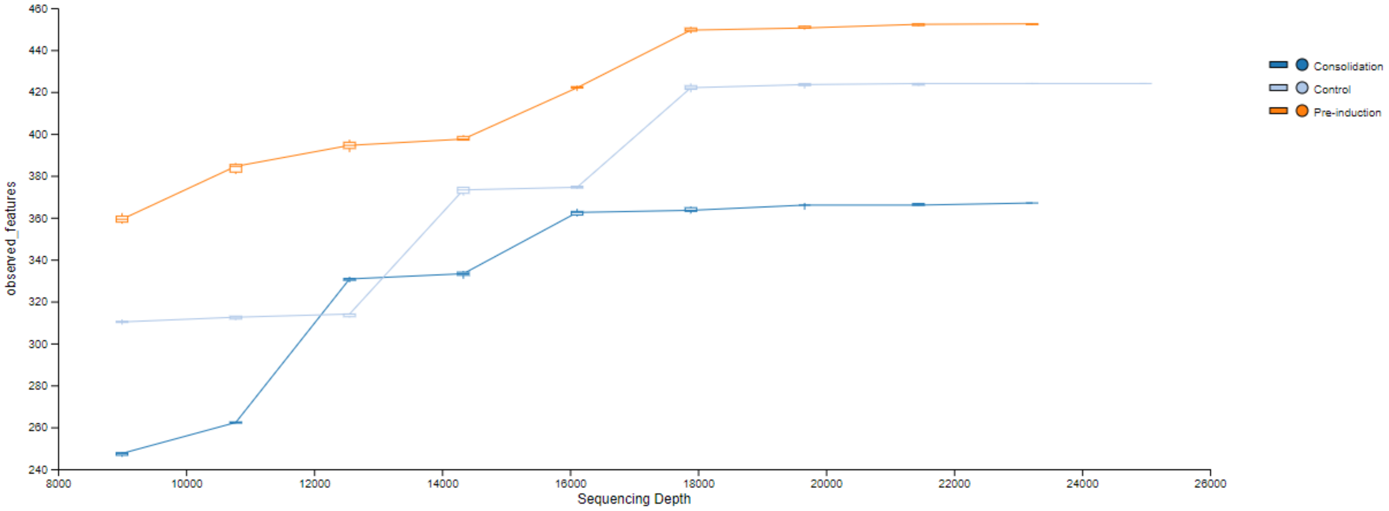

Figure S1 – The figure S1 shows rarefaction curves for groups (Consolidation, Control and Pre-induction).
